# Training recollection in healthy older adults: clear improvements on the training task, but little evidence of transfer

**DOI:** 10.3389/fnhum.2014.00898

**Published:** 2014-11-18

**Authors:** Vessela Stamenova, Janine M. Jennings, Shaun P. Cook, Lisa A. S. Walker, Andra M. Smith, Patrick S. R. Davidson

**Affiliations:** ^1^Rotman Research Institute, Baycrest – University of TorontoToronto, ON, Canada; ^2^Department of Psychology, Wake Forest UniversityWinston-Salem, NC, USA; ^3^Department of Psychology, Millersville UniversityMillersville, PA, USA; ^4^School of Psychology, Faculty of Social Sciences, Ottawa Hospital Research Institute, University of OttawaOttawa, ON, Canada

**Keywords:** aging, memory, rehabilitation, recollection, familiarity

## Abstract

Normal aging holds negative consequences for memory, in particular for the ability to recollect the precise details of an experience. With this in mind, Jennings and Jacoby (2003) developed a *recollection training* method using a single-probe recognition memory paradigm in which new items (i.e., foils) were repeated during the test phase at increasingly long intervals. In previous reports, this method has appeared to improve older adults’ performance on several non-trained cognitive tasks. We aimed to further examine potential transfer effects of this training paradigm and to determine which cognitive functions might predict training gains. Fifty-one older adults were assigned to either recollection training (*n* = 30) or an active control condition (*n* = 21) for six sessions over 2 weeks. Afterward, the recollection training group showed a greatly enhanced ability to reject the repeated foils. Surprisingly, however, the training and the control groups improved to the same degree in recognition accuracy (d′) on their respective training tasks. Further, despite the recollection group’s significant improvement in rejecting the repeated foils, we observed little evidence of transfer to non-trained tasks (including a temporal source memory test). Younger age and higher baseline scores on a measure of global cognitive function (as measured by the Montreal Cognitive Assessment tool) and working memory (as measured by Digit Span Backward) predicted gains made by the recollection training group members.

## INTRODUCTION

Two broad approaches have been taken in cognitive rehabilitation of episodic memory. The first is to train people to use various strategies for memory encoding and/or retrieval. Although older adults can benefit from such training (for review, see Glisky and Glisky, 2008), many strategies are only appropriate for certain types of materials and/or relationships (e.g., in learning associations between faces and names), and once people are taught such strategies they are then left to apply them appropriately to daily life. The second broad approach is to attempt to repair or improve the cognitive processes that underlie episodic memory (for example see Buschkuehl et al., 2008; Brehmer et al., 2012). A major concern regarding this approach is generalization: Although it is relatively easy to produce improvements on the particular tasks being trained, it is more difficult to produce convincing evidence of improvement on tasks that were not trained but that depend on the cognitive process(es) that underwent training (e.g., Owen et al., 2010; Ranganath et al., 2011). In this paper we explored a potentially fruitful technique developed by Jennings and Jacoby (2003), which they argued can improve memory in healthy older people by focusing on recollection.

Dual-process theories propose that both recollection and familiarity can influence memory (Mandler, 1980, 2008; Jacoby, 1991; Yonelinas, 2002). *Recollection* can be thought of as the essence of episodic memory: a vivid re-experiencing of the details of the encoding event (i.e., the “what,” where,” and “when” of the event; Tulving, 2002). According to several theories, recollection often contributes significantly to performance on tests of recognition memory, and may contribute even more heavily to performance on tests of recall (Quamme et al., 2004; McCabe et al., 2011).

In contrast, *familiarity* entails a more general feeling that a stimulus or an event has been experienced in the past, and may contribute less strongly to recall than it does to recognition performance (McCabe et al., 2011). Aging is often associated with a decline in recollection, whereas familiarity remains relatively undisrupted (or, at least, can vary from study to study; for review, see Light, 2012).

One piece of evidence of recollection’s decline in aging comes from Jennings and Jacoby’s (1997) “opposition procedure.” On this task, which places recollection and familiarity in opposition to one another, the participant is given a study list, followed by a recognition test in which both old and new words appear. The participant’s goal is to endorse only those items that had appeared on the study list. The critical words on the task are the new words (i.e., foils), because these are shown *twice* during the test. On its second presentation, a repeated foil feels more familiar but must still be rejected. The only way to correctly do so is to recollect the context in which the word was seen previously (i.e., participants should remember that the word only appeared in the test list, and *not* in the study list.). If participants rely on familiarity alone during this decision, they will erroneously endorse the repeated foil and commit what Jennings and Jacoby (1997) call a *repetition error*. Older adults are much more likely to commit repetition errors than are young adults (Jacoby, 1999; Benjamin, 2001).

### RECOLLECTION TRAINING WITH THE REPETITION-LAG PARADIGM: PROMISING PRELIMINARY RESULTS

To improve recollection in older adults, Jennings and Jacoby (2003) modified the opposition procedure by steadily lengthening the repetition lag (i.e., the interval over which foils were repeated during the test) across several training sessions. Before undergoing this “recollection training,” the older adults frequently made repetition errors, even when only a few words intervened between the first and second presentation of a given foil. After a week of training, however, the older adults could perform the task very well, avoiding repetition errors even when 28 items intervened between the first and second presentation of a foil. This improvement was observed only in the experimental group, in which the repetition lag was gradually lengthened for each participant once he or she had achieved a certain level of performance (i.e., the *adaptive* training group); little improvement was seen in a yoked active control group in which different length lags were presented randomly across training days (i.e., non-adaptive).

What makes this paradigm so interesting is that it is one of the few existing cognitive rehabilitation methods to show the potential to ameliorate recollection deficits in older adults and transfer those gains to non-trained tasks. Jennings et al. (2005) categorized their transfer tasks into *near-transfer* versus *far-transfer* on the basis of the underlying processes involved. For example, verbal n-back (Dobbs and Rule, 1989; Jonides et al., 1997), self-ordered pointing (Petrides and Milner, 1982) and source discrimination were designated as near-transfer because they all involved retrieval of contextual information (temporal, output, and source monitoring, respectively). Digit symbol substitution (Wechsler, 1997), reading span (Daneman and Carpenter, 1980), and verbal free recall [as assessed using the California Verbal Learning Test-Second Edition (CVLT-II); Delis et al., 2001] were categorized as far-transfer, due to the fact that they were considered to have different underlying processes. Their recollection training group improved mostly on the near transfer tests: one- and two-back working memory, self-ordered pointing, and source discrimination with far transfer gains seen only on digit symbol substitution. There were no significant changes on the three-back task or on the CVLT-II.

Other studies have replicated the benefits of this method to some of the same non-trained tasks used by Jennings et al. (2005): digit symbol substitution (Bailey et al., 2011) and self-ordered pointing (Bailey et al., 2011; but see Lustig and Flegal, 2008 for exception) in older adults, and 2-back working memory, and source discrimination in Alzheimer’s patients (Boller et al., 2012). Transfer effects have also been reported to Trail Making Part B (Lustig and Flegal, 2008), and the AX-CPT task (Quamme et al., 2004) in older adults (Bailey et al., 2011) and delayed matching-to-sample in Alzheimer’s patients (Boller et al., 2012). Curiously, Boller et al. (2012) failed to observe transfer effects to reading span, free recall, or recognition memory in Alzheimer’s patients [recognition memory did improve in Boller et al. (2012), but did so in both the recollection training and the control training group].

### THE PRESENT STUDY

Based on this previous work, we sought to answer two major questions:

#### Does recollection training produce clear improvements on non-trained memory tasks?

As reviewed above, past studies have shown evidence of transfer to non-trained tasks, although not universally (Jennings et al., 2005; Lustig and Flegal, 2008; Boller et al., 2012). One of the reasons for variability among the previous studies may be that they have used relatively small groups (*n*s = 12 in Boller et al., 2012; *n*s = 12–17 in Jennings et al., 2005). In the present study we aimed to examine a number of measures that, based on past studies, appeared likely to show transfer, and to use larger sample sizes than previously, to increase statistical power. The strongest evidence of recollection training yielding a general benefit to memory would come from a comparison of two groups (recollection training versus a well-matched active control group with equal training gains expectations as recommended by Boot et al., 2013), assessed on non-trained tasks before and after training. Any improvements shown by the recollection training group above-and-beyond those seen in the active control group (in the form of a significant group by time interaction, Nieuwenhuis et al., 2011) could be attributed to the training, rather than to non-specific factors such as improvement in mood or comfort/reduction in stress, benefits of social/intellectual stimulation, and/or expectations of improvement (i.e., placebo).

We organized our non-trained measures by sorting them into those on which we should be most likely to see transfer effects, versus those that we should be less likely to see transfer. At the closest end of the spectrum, we designated two of our non-trained measures as “near transfer”: temporal order memory from an experimental source monitoring paradigm, and total across list intrusions on Long Delay Free Recall from the CVLT-II, both of which require participants to remember the conjunction between a specific item and the temporal context in which it appeared, which is essentially what the training in the Jennings and Jacoby (2003) paradigm involved.

We included several other “intermediate transfer” scores, all of which reflect episodic memory, and all of which we expected might show improvement based on theoretical and/or empirical grounds (Bailey et al., 2011; Boller et al., 2012). In addition to temporal order memory, our experimental source monitoring paradigm included measures of perceptual (i.e., voice) and spatial (i.e., location) source. Given these measures are measures of source memory, we considered them to be close to the contextually rich recollection memory that was being trained and thus we classified them as “intermediate transfer.” The CVLT-II Long Delay Free Recall scores were also considered “intermediate transfer” as we were working under the assumption that recollection supports recall (McCabe et al., 2011). Jennings et al.’s (2005) recollection-training group showed numerically improved CVLT-II Long Delay Free Recall scores from baseline to post-training (improving from 69 to 75%). We hypothesized that a lack of power may have resulted in this numerical difference not attaining statistical significance and that with larger groups we would see significantly greater improvements on these measures in the recollection training group than in the active control group.

**FIGURE 1:**
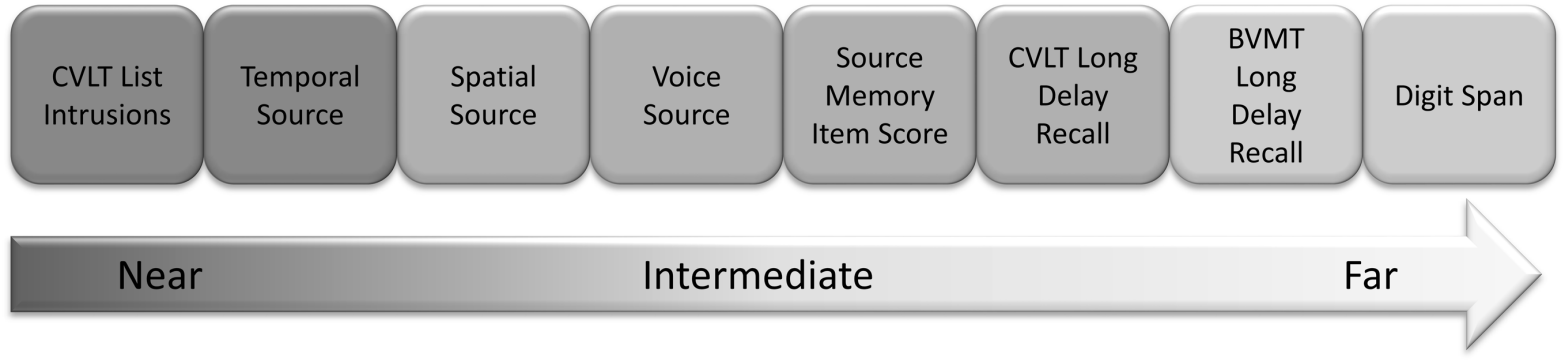
**Near-, intermediate- and far-transfer measures**.

The Brief Visuospatial Memory Test-Revised (BVMT-R; Benedict, 1997) was considered “far transfer,” given the different modality (visual as opposed to the verbal memory that was being training in the recollection-training task). Finally, given the above-listed evidence that recollection training might also improve performance on working memory tasks (verbal n-back, self-ordered pointing), we included a short-term/working memory measure: Digit Span (from the Wechsler Adult Intelligence Scale-III (WAIS-III), Wechsler, 1997), which has been shown in the past to correlate with n-back (Jaeggi et al., 2010). We considered this test to be a measure of “far-transfer”, given it does not require the support of long-term memory. The ranking of all transfer measures is displayed in **Figure 1**. Finally, we included the Multifactorial Memory Questionnaire (MMQ, Troyer and Rich, 2002), to examine whether participants’ self-perceptions and reported strategies might change from before to after training.

In addition to the group comparisons, we also asked whether, on an individual-by-individual basis, the gains made in training relate to the gains made on the transfer tasks. We were especially interested in whether those who show greater gains on the training task would also show greater gains on the transfer measures.

#### Do those who come into the training with better memory improve to a different degree from those with poorer memory?

In previous work with the recollection training paradigm, clearly some participants have improved more than others: For example, of Jennings and Jacoby’s (2003) *n* = 12 recollection training participants, three showed barely any advancement, three reached the maximum lag by the end of training, and the rest fell in between. In an attempt to explain these individual differences, Bissig and Lustig (2007) ranked older adults by how much they improved on a modified version of the recollection training paradigm. Those who were younger and had higher verbal intelligence (as measured by a vocabulary test) showed greater recollection training gains. Note, however, that Bissig and Lustig (2007) modified the original method to allow for self-paced encoding. For this reason, we asked whether similar results would be obtained with the original training paradigm. Given previous reports that cognitive training tends to be more beneficial for individuals with higher cognitive function (Yesavage et al., 1990), our hypothesis was that people with better cognitive function may benefit more from the training. This is similar to the so called “Matthew Effect” proposed in the developmental literature by Stanovich (1986) who proposed that children who have better reading abilities early on tend to develop better reading and learning skills later in life, while children with lower reading abilities early on develop poorer reading skills. Higher cognitive function may also be related to higher cognitive reserve and participants with higher cognitive reserve may benefit more from cognitive training (Stern, 2002).

## MATERIALS AND METHODS

### PARTICIPANTS

Forty-three older adults were randomly assigned to either a *recollection training* (*n* = 22) or an active *recognition control* group (*n* = 21). At the end eight more participants were assigned directly to the recollection training condition, to increase its size to *n* = 30 (these participants did not differ from the rest of the sample on any of the variables the participant groups were matched on). The study was single-blind: participants were not aware of which condition they were in, although the examiner (V. S.) was. The sample size was based on Jennings et al.’s (2005) “repetition-lag” recollection training study which included 17 participants each in the recollection and in the recognition control group. Their treatment effect, as measured by the change in lag level from the beginning to the end, was large (Cohen’s *d* = 1.79). The participants were living independently in the community (Ottawa, ON, Canada) and recruited through local newspaper ads. There were no statistically significant group differences (see **Table 1**) in age, education, sex, handedness, cognitive status based on the Montreal Cognitive Assessment, (MoCA; Nasreddine et al., 2005) and mood as measured by the Center for Epidemiological Studies Depression scale (CES-D). The last scale was included after the beginning of the study, so the first nine participants did not complete it. MoCA scores ranged from 21 to 30. Although a few participants scored below the MoCA’s official recommended cut-off of 26, a more recent study indicates that a cut-off of 20 may be more appropriate, and for this reason we kept all participants (Waldron-Perrine and Axelrod, 2012). For the CES-D, we used a cut-off score of 27, which has been recommended for older adults (Schein and Koenig, 1997). Only one participant exceeded this level with a score of 31, but she reported that her 60th birthday had occurred that week and had made her feel worse than normal, and she asserted that she was not depressed. A number of participants (*n* = 12) indicated having ongoing mental health problems, mostly depression which was successfully treated. To ensure that these participants did not significantly affect our results, we re-ran all analyses without them and our pattern of results did not change in any way.

**Table 1:** Participants’ demographic information.

	Recognition control	Recollection training	
	*M (SD)*	*M (SD)*	*p*-value
Age (years)	68.62 (6.39)	67.60 (5.56)	0.55
Education (years)	16.57 (2.99)	15.57 (3.09)	0.25
Sex (F/M)	19/2	23/7	0.28
Handedness (R/L/A)	19/1/1	29/0/1	0.46
MoCA (/30)	27.24 (2.48)	25.97 (2.52)	0.09
CES-D^a^	9.61 (7.72)	8.08 (5.69)	0.46

*MoCA, Montreal Cognitive Assessment; CES-D, The Center for Epidemiological Studies Depression scale.*

*^a^Sample size (*n* = 21, *n* = 30).*

### MATERIALS AND PROCEDURE

The project was approved by the University of Ottawa Research Ethics Board and all participants provided informed consent before taking part.

#### Training overview

Participants came in for three training sessions per week for 2 weeks. Each training day, they completed four sessions (each session involved studying one list and then performing the corresponding recognition memory test). Participants usually completed the four training sessions in 20–30 min. This schedule was similar to the one used in recollection training of older adults (Jennings et al., 2005) and identical to the one used with Alzheimer’s patients (Boller et al., 2012).

#### Training tasks

**FIGURE 2:**
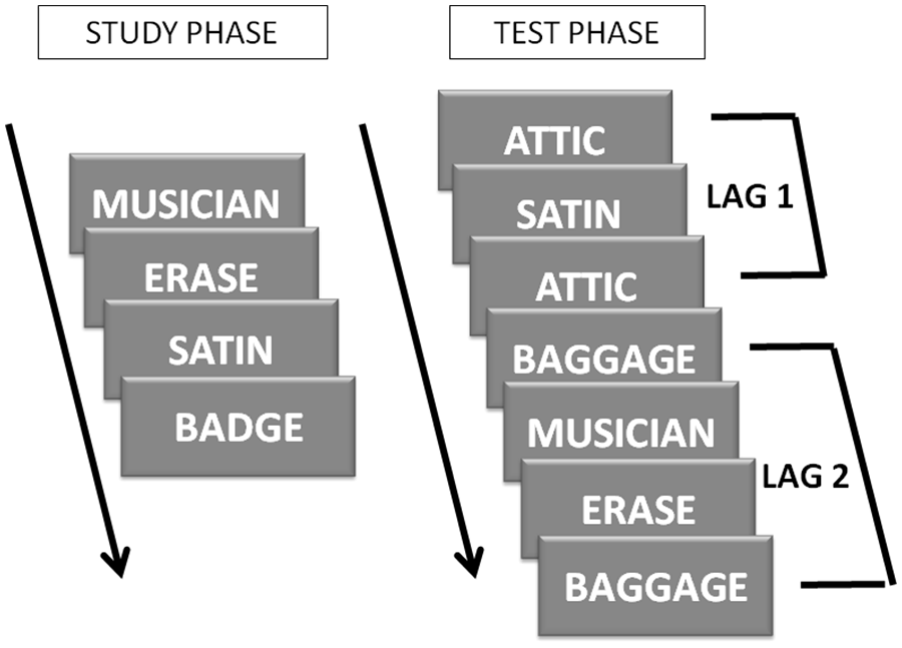
**Recollection training design.** During the study phase, words were presented one at a time in the middle of the screen. During the test phase studied words and new words were presented. New words were repeated at two lag intervals. All participants started at lags 1 and 2, which is schematically represented above.

***Recollection training.*** The task was identical to that used by Jennings et al. (2005). Briefly, participants studied lists of 30 words presented one at a time for a 2-s period and were asked to read each word out loud and try to commit it to memory. Each study list was followed by a recognition test, which included the studied items plus 30 new items (each shown twice and repeated at a specific lag interval) and five “filler” words which were necessary to complete the interval lags. The words were nouns, verbs, and adjectives balanced across each list for frequency of occurrence in the language. There were 128 study word, 128 new word, and 128 filler word lists. On the test, participants were asked to respond to words they could remember having read on the study list with “Yes” and to words that they could not remember from the original list with “No” by pressing one of two keys on the keyboard. The instructions also stated that when the participant was correct the word “CORRECT” would appear on the screen, but when the participant was not correct, nothing would appear. Finally, they were informed that some of the new words might reappear during the test but that they were still to say “No” to them, because they were not study list words; only new words would reappear during the test, whereas old (i.e., study list) words would appear only once during the test. In addition, they were asked to use the feedback they received to improve their performance if possible. The new words were split into two groups and each group of words was repeated at one of two different lag intervals. The lag interval pairs were 1 and 2; 1 and 3; 2 and 4; 2 and 8; 4 and 12; 4 and 16; 8 and 20; 8 and 24; 12 and 28; 12 and 32; 16 and 36; 16 and 40; 20 and 44; 20 and 48; and 24 and 52. All participants started their first set at lag 1 and 2, which means that half of the new words presented at the test were repeated at lag 1 and half were repeated at lag 2 (**Figure 2**). Participants had to reach a performance criterion at both lag levels in order to be moved up a level. The criterion was a maximum of one repetition error in identifying the repeated items for lags 1–4, and two repetition errors for lags 8–52. By having two different lag intervals in each run, one ensures that the participants always work at one lag interval level that they have mastered already. Participants had to meet criterion at *both* lag intervals in order to move to the next level. If participants did not achieve criterion at both lags, they remained at the same lag level for the next session and stayed at that level until they met criterion.

***Active recognition control task.*** A single-probe verbal recognition task was designed as an active control condition using exactly the same words as on the recollection training task. Here participants studied 30 words, but on the recognition test the new words were not repeated. Instead, 65 new words were randomly intermixed with the 30 old words. Participants were instructed to press one of two keys to indicate their Yes/No response for each test item and they were also given feedback when they were correct in the same manner as the recollection training task. These participants came in on the same schedule as the other group (i.e., three times per week for 2 weeks, performing four study sessions per visit, which took the same amount of time as the recollection training did). This control condition was considered to be superior to the one used previously by Jennings et al. (2005), in that it used exactly the same study list length and identical word lists as in the recollection training.

#### Transfer tasks

***California Verbal Learning Test-Second Edition (Delis et al., 2001).*** Participants studied 16 words (list A) that fell into one of four different categories. The list was read at a pace of one word per second and then participants were asked to recall as many words as possible. This was repeated four times. A second list (List B) of 16 words falling into four categories (two of these categories being the same as those on List A) was read. Participants were again asked to recall the list. This was followed by List A Short Delay Free and then Cued Recall. After a 20 min filled delay interval Long Delay Free Recall for List A was administered, followed by Long Delay Cued Recall for List A, and, finally, a yes-no recognition test for List A.

***Brief Visuospatial Memory Test-Revised (Benedict, 1997).*** Participants were shown a page with six geometrical figures (2 per row) for 10 s and asked to commit them to memory (including the spatial location of the figures). They were then asked to draw them on a blank sheet of paper immediately afterward, drawing the figures as accurately as they could and placing them in the appropriate location on the page. This same procedure was performed two more times. After the third trial, participants were reminded not to forget the stimuli, because they might be asked to draw them later from memory. After a 25 min delay, the participants were asked to draw them one more time. After this delayed recall, participants were given a recognition test with six old and six new items and asked to respond by saying ‘Yes’ to the old items and ‘No’ to the new items.

***Wechsler Adult Intelligence Scale-III (WAIS-III) Digit Span Forward/Backward (Wechsler, 1997).*** Digits were read at a one digit per second rate and participants were asked to repeat them either exactly as they were read to them (Digit Span Forward) or in backward order (Digit Span Backward).

***Source memory.*** We administered a task developed by Cook (Cook, 2007; Davidson et al., 2013). One hundred and sixty sentences were recorded by three native English speakers, one female and two males. The sentences were emotionally neutral (“She put the rice on to boil and set the time to 20 minutes”). The task consisted of four different conditions: voice source, spatial source, temporal source, and item memory. The voice, spatial, and temporal source conditions each contained two practice items and 16 test items. The item memory test contained two practice items and 40 test items. In all conditions, as the participants were presented with the sentences they were asked to rate on a 5-point scale (‘very likely,’ ‘likely,’ ‘no opinion,’ ‘unlikely,’ and ‘very unlikely’) how likely it is that the sentence would be heard on the radio. For the spatial source condition, all sentences were spoken by the same voice but were presented either on the left or on the right side through a loudspeaker and participants were asked to pay attention to the location because they were to be tested on this later. In the voice source condition, half of the sentences were spoken by a male and the other half by a female voice, and participants were asked to commit to memory which sentences were spoken by the man and which by the woman. In the temporal source condition, all sentences were spoken by a male and a single bell was rung halfway through the list. Participants were asked to indicate which sentences occurred before and which sentences occurred after the bell. For both the voice and temporal source conditions, the sentences were presented using the left and right speakers. Finally, for the item memory test, participants were asked to commit to memory all the sentences as best as they could. Each condition involved a forced choice recognition test. For the spatial, voice, and temporal source tests, participants were shown a sentence written on the screen and asked to indicate whether it had originally been presented in the Left/Right, Male/Female, or Before/After the bell context for each condition respectively by a key press. For the item memory test, participants were presented with pairs of sentences and asked to indicate by a key press which one of the two was a sentence that they had originally heard.

There were six different ways in which the administration of the source memory conditions were ordered (administration order). These orders ensured that each source memory task condition (spatial, temporal, or voice) appeared in each position (i.e., first, second, third) and that each source memory task condition was preceded and followed the same number of times by each of the other source memory task conditions. Item memory blocks were always last. Overall, there were eight sentence lists, which were rotated through the six administration orders to ensure that each sentence list was used at least once as targets for each source condition, as targets for the item condition, and as distractors for the item condition. This means there was some overlap in sentence lists between pre- and post-training for a given participant, but sentences that were present in both pre- and post-training versions never appeared in the same source memory condition. All sentences were presented using DMDX display software Version 4.0.6.0 (Forster, 2012).

***The Multifactorial Memory Questionnaire (MMQ; Troyer and Rich, 2002).*** We included this questionnaire to assess self-reports on three dimensions of memory: contentment or satisfaction with one’s own memory ability (MMQ-Contentment), perception of everyday memory ability (MMQ-Ability), and use of everyday memory strategies and aids (MMQ-Strategy). MMQ-Contentment includes 18 statements (e.g., ‘I have confidence in my ability to remember things’) each with one of five options for endorsement: Strongly Agree, Agree, Undecided, Disagree, and Strongly Disagree. MMQ-Ability includes 20 descriptions of abilities or problems (e.g., ‘Not recall the name of someone you have known for some time.’) with the following response options: All the time, Often, Sometimes, Rarely, and Never. Finally, MMQ-Strategy includes 19 descriptions of various strategies the participant may be using (e.g., ‘Write things on a calendar, such as appointments or things you need to do.’) and response options were the same as in the MMQ-Contentment. Participants filled out the questionnaires themselves by checking one of the five options beside each statement.

***Administration of transfer tasks.*** All transfer tasks were administered before and after the training. In most cases the pre-training session took place the week before the training started (i.e., Week 1), the training was conducted during Weeks 2 and 3, and the post-training session was completed the week after the training was completed (Week 4). All but three participants completed the study in a quiet, well-lit testing room at University of Ottawa (three participants in the recollection training group preferred to be tested at home). All participants were administered a general demographic and health questionnaire and then proceeded to the cognitive tests in the following order: (1) CVLT-II: five trials of List A Immediate Free Recall, (2) CVLT-II: List B, (3) CVLT-II: Short Delay Free Recall, (4) CVLT-II: Short Delay Cued Recall, (5) BVMT-R: three learning trials, (5) MMQ, (6) Digit Span Forward/Backward, (7) CVLT-II: Long Delay Free Recall, (8) CVLT-II: Long Delay Cued Recall, (9) CVLT-II: Recognition, (10) BVMT: Delayed Recall, (11) BVMT: Recognition, (12) MoCA, (13) source memory. The CVLT-II has two parallel versions, which were administered in counterbalanced order at each session. The BVMT-R has six versions; only versions 1 and 2 were administered and those were counterbalanced across the two assessments. The same version of the digit-span test was administered before and after training. Participants were administered different source memory administration order versions before and after (if on their first assessment they were administered version 1, after training they were administered version 2, if administered 2 at pre-training they were administered version 3 at post-training, and so on). All were counterbalanced across participants.

## RESULTS

### TRAINING

#### Recollection training progress

**FIGURE 3:**
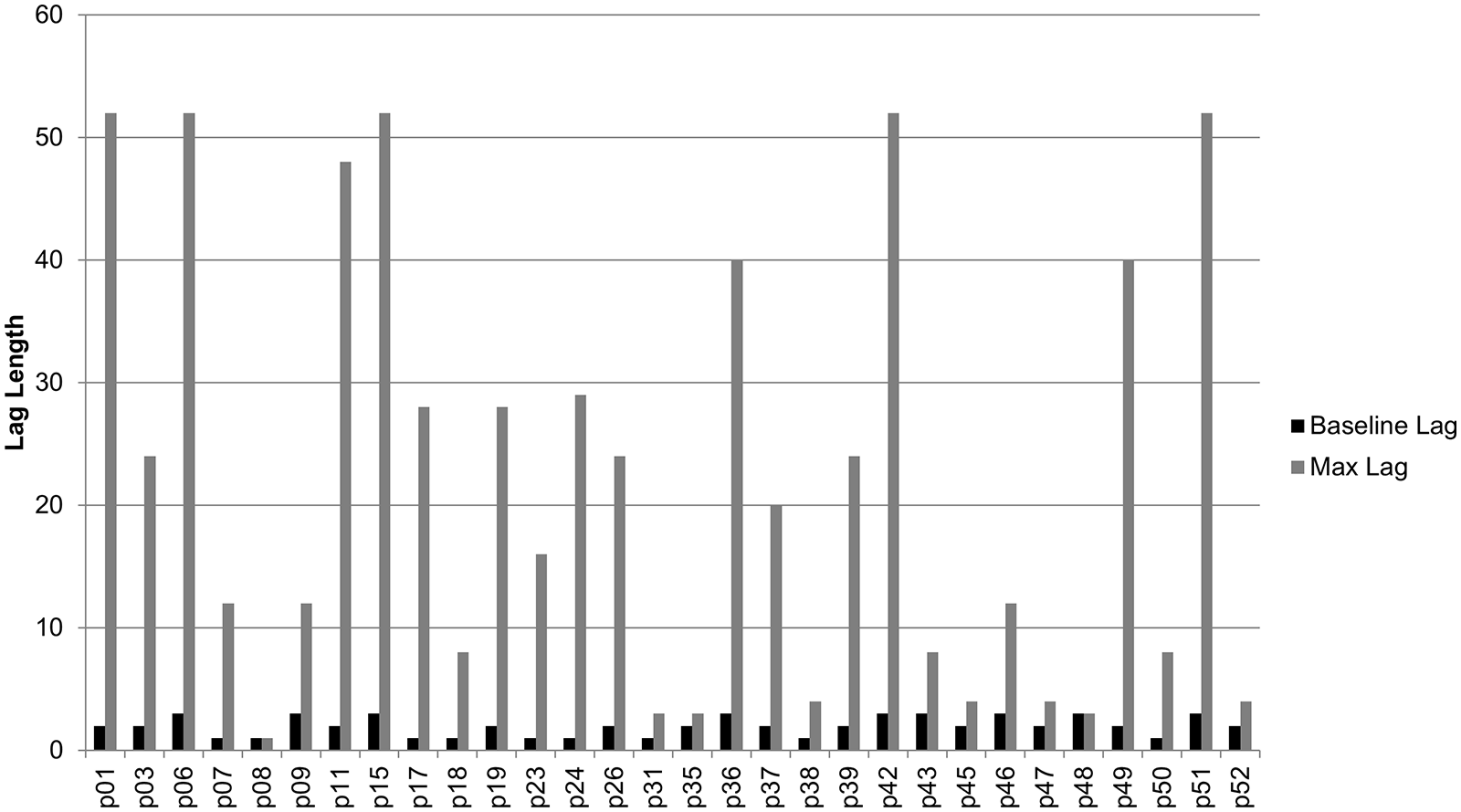
**Baseline vs. maximum lag reached by each participant in the recollection training group**.

To determine progress through the training, we compared the longest lag interval reached by Session 3 on Day 1 (to give us a sense of baseline abilities) against the longest lag interval achieved by the end of the training (i.e., session 24, Day 6) following Jennings et al. (2005). These Day 1 scores did not appear to be obscured by a ceiling effect: Only one participant progressed as rapidly as possible through the initial (short) lags – all the others made repetition errors initially. A repeated measures *t*-test showed a significant improvement from the baseline lag interval reached by Session 3 on Day 1 (*M* = 2.00, SD = 0.79) to the maximum lag interval reached by the end of training (*M* = 21.53, SD = 18.43), *t*(29) = 5.92, *p* < 0.001, *d* = 1.96 (**Figure 3**).

#### Recognition memory on the training and control tasks

**FIGURE 4:**
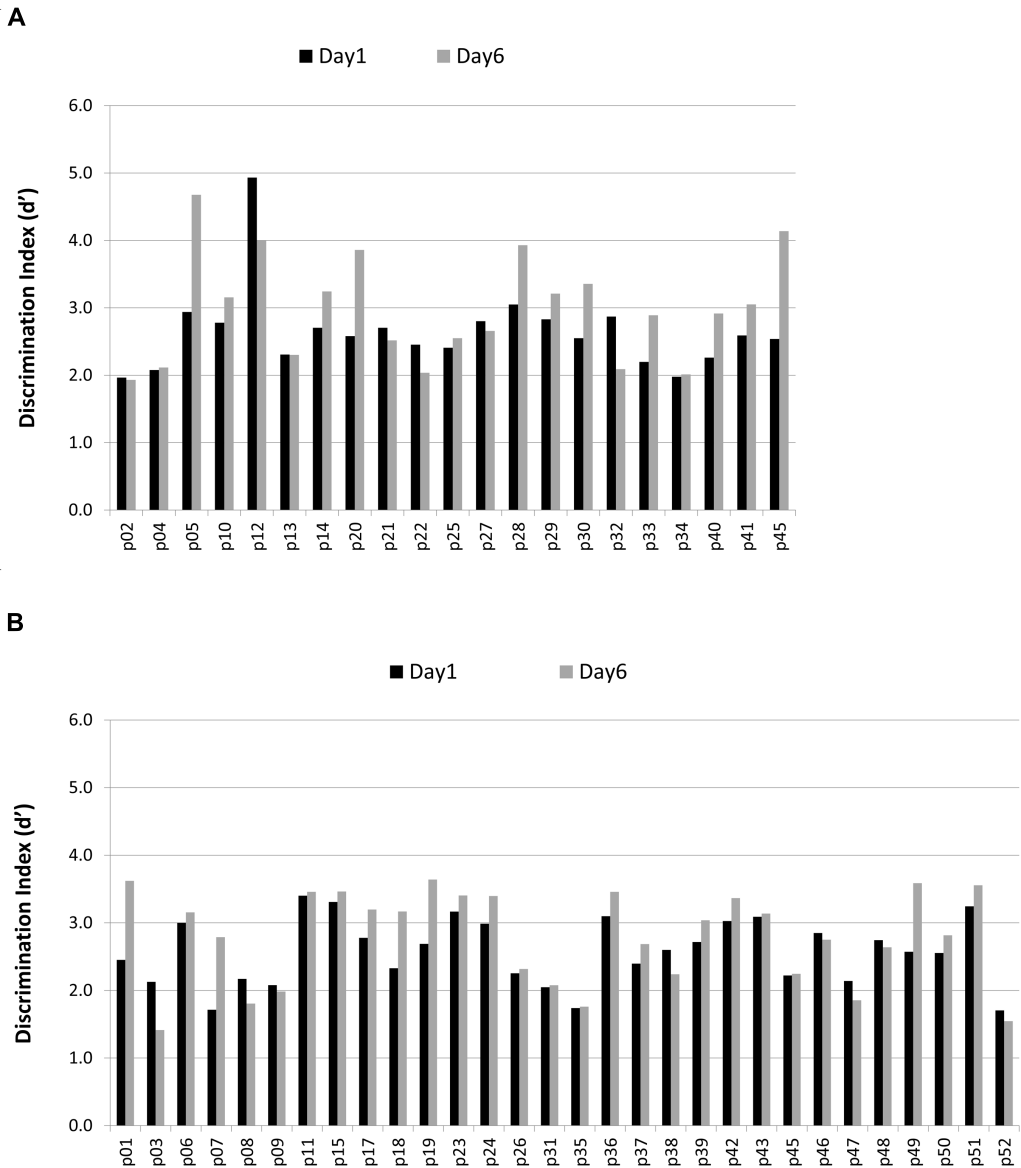
**Discrimination Index for each participant in **(A)** the recognition control group and **(B)** the recollection training group on Day 1 and Day 6 of training**.

To assess recognition memory, we calculated the probability of responding “yes” to studied items (hits) and to new items on their first presentation (new false alarms). For both scores, we compared the average performance on Day 1 (collapsed across the four sessions that day) against the average performance on Day 6 (collapsed across the four sessions that day). Hit and false alarm rates and discrimination (d′) and response bias (C; Snodgrass and Corwin, 1988) indices are listed in **Table 2**. Please refer to **Figure 4** for individual participant changes in discrimination index in each group. We conducted a 2 × 2 [Group (Recollection training, Recognition control) × Time (Beginning, End)] mixed analysis of variance (ANOVA) for each of these four variables. Hits and false alarms were corrected by adding 0.5 to the number of hits (or false alarms respectively) and dividing by the number of items +1, to correct for situations in which scores were at ceiling or at floor (as per Snodgrass and Corwin, 1988).

**Table 2:** Hit and false alarms rates, and discrimination index (d′) and response bias (C) at Day 1 and Day 6 of training.

	Recognition control		Recollection training	
	Day 1 *M (SD)*	Day 6 *M (SD)*	Day 1 *M (SD)*	Day 6 *M (SD)*
Hit rate	0.86 (0.08)	0.87 (0.07)	0.78 (0.1)	0.77 (0.12)
False alarm rate	0.09 (0.04)	0.06 (0.04)	0.11 (0.07)	0.07 (0.06)
Discrimination (d′)	2.53 (0.49)	2.83 (0.68)	2.26 (0.53)	2.50 (0.74)
Bias (C)	0.15 (0.21)	0.20 (0.15)	0.27 (0.27)	0.45 (0.21)

The recognition control group obtained an overall higher *hit rate* than the recollection training group, *F* (1,49) = 12.95, *p* = 0.001, η^2^ = 0.21. There was no significant time effect, *F*(1,49) < 0.001, *p* = 0.99, η^2^ = 0 or interaction, *F*(1,49) = 2.07, *p* = 0.16, η^2^ = 0.04.

Both groups decreased significantly in their *false alarm* rates, *F*(1,49) = 14.81, *p* = 0.0003, η^2^ = 0.23 from the beginning to the end of training, but no group *F*(1,49) = 0.73, *p* = 0.40, η^2^ = 0.02 or interaction effects, *F*(1,49) = 1.21, *p* = 0.27, η^2^ = 0.02 were observed.

Both groups improved in their *discrimination (d*′*)* over time *F*(1,49) = 13.70, *p* = 0.001, η^2^ = 0.22. No group, *F*(1,49) = 3.57, *p* = 0.07, η^2^ = 0.07, or interaction effect, *F*(1,49) = 0.12, *p* = 0.73, η^2^ = 0.002, was observed.

Finally, both groups became more conservative in their responses over time, *F*(1,49) = 9.46, *p* = 0.003, η^2^ = 0.16 and overall the recollection training group was more conservative than the recognition control group, *F*(1,49) = 14.14, *p* = 0.0005, η^2^ = 0.22. The degree of change did not differ significantly between the two groups, however: interaction *F*(1,49) = 2.77, *p* = 0.11, η^2^ = 0.05.

#### Predictors of training gains

As shown in **Figure 3** participants varied considerably in their progress through the training. Using the method of Bissig and Lustig (2007), we ranked participants based on their gains made during training. Participants were first ranked based on the longest lag level achieved (e.g., a lag of 8 was ranked lower than a lag of 12 with higher rank indicating larger gains in training). Ties were broken based on when the longest lag was achieved, with participants reaching their longest lag earlier being ranked higher than participants reaching it later (e.g., reaching lag 40 by session 23 was ranked higher than reaching it by session 24). Finally, any remaining ties were broken based on accuracy of performance on the repeated words, with participants with higher accuracy ranked higher.

Following Bissig and Lustig (2007), we conducted a hierarchical regression using rank–ordered training gains as the dependent variable. Given, Bissig and Lustig (2007) showed that two demographic characteristics, age and vocabulary, served as reliable predictors of rank, we entered age and years of education into the first model. Measures of cognitive status at the time of testing were entered in the second model and included the baseline MoCA score, the percentage accuracy baseline scores of the CVLT-II Long Delay Free Recall, and the Digit Span Forward and Backward as the independent variables. The Source Memory score was excluded, because fewer participants completed the Source memory tasks (i.e., the sample size was smaller) due to technical issues with the task and inability to administer the task at a participant’s home if s/he was assessed there (the number of participants in each source task are listed in **Table 3**). Using a hierarchical regression allowed us to examine the importance of demographic variables in predicting rank and estimate the amount of variability explained by the cognitive status of the participants over and above that explained by the demographic variables. The regression results are summarized in **Table 4**; Multiple R for the first block of regressors (age and YOE) was close to statistical significance, *F*(2,29) = 2.96, *p* = 0.069; multiple R for the next block of regressors was significant *F*(6,29) = 3.12, *p* = 0.022. The demographic variables (Age and YOE) explained 18% of the variance, while adding the cognitive status scores in block 2 of the analysis increased the amount of variability explained to 45%. This increase is significant by the F change test *F*(4,23) = 2.81, *p* = 0.049. Among the demographic variables, only age was significant, while among the cognitive status, Digit Span Backward (*p* = 0.049) was statistically significant, followed by the MoCA (*p* = 0.098), which was marginally significant.

**Table 3:** Pre- and post- training scores on transfer measures.

	Recognition control		Recollection training		*p*-value		
	Pre *M* (SD)	Post *M* (SD)	Pre *M* (SD)	Post *M* (SD)	Group	Time	Group × time
CVLT-II list 1–5 proportion accuracy^a^	0.77 (0.10)	0.76 (0.13)	0.70 (0.15)	0.69 (0.18)	0.059	0.75	0.84
CVLT-II SD free proportion accuracy^a^	0.79 (0.17)	0.82 (0.18)	0.71 (0.22)	0.72 (0.25)	0.11	0.38	0.80
CVLT-II LD free proportion accuracy^a^	0.82 (0.18)	0.84 (0.19)	0.78 (0.21)	0.79 (0.23)	0.34	0.61	0.96
CVLT-II total across list intrusions	0.05 (0.22)	0.86 (1.31)	0.86 (1.90)	1.03 (2.47)	0.24	0.11	0.29
BVMT-R T1–3 proportion accuracy	0.69 (0.21)	0.71 (0.21)	0.71 (0.11)	0.72 (0.13)	0.70	0.46	0.97
BVMT-R DR proportion accuracy	0.83 (0.15)	0.84 (0.14)	0.83 (0.13)	0.83 (0.13)	0.94	0.73	0.95
Digit Span Forward^b^	10.60 (2.01)	10.20 (1.40)	10.23 (1.72)	10.70 (1.74)	0.89	0.87	0.04*
Digit Span Backward^b^	7.45 (2.42)	8.90 (2.36)	7.23 (2.33)	7.97 (2.63)	0.35	0.003**	0.31
Source memory spatial %^c^	62.50 (15.31)	59.19 (14.20)	58.15 (13.25)	67.39 (12.10)	0.54	0.34	0.047*
Source memory temporal %^d^	66.91 (15.11)	64.33 (11.43)	63.10 (13.67)	65.77 (17.52)	0.09	0.99	0.36
Source memory voice %^d^	72.79 (16.82)	77.57 (17.12)	75.60 (13.82)	73.51 (17.66)	0.89	0.61	0.20
Source memory item %^d^	94.26 (4.40)	93.24 (7.22)	92.07 (9.22)	90.87 (10.99)	0.37	0.35	0.94
MMQ contentment (/72)	39.33 (12.08)	42.38 (10.77)	40.73 (12.00)	41.73 (9.87)	0.90	0.10	0.40
MMQ ability (/80)	46.1 (9.20)	47.60 (9.28)	48.00 (10.78)	49.41 (10.72)	0.49	0.24	0.97
MMQ strategy (/76)	41.10 (8.74)	38.0 (7.21)	35.93 (10.92)	35.20 (8.31)	0.09	0.14	0.36

CVLT-II List 1–5, average proportion accuracy on Trials 1–5; CVLT-II SD, CVLT-II short. delay; CVLT-II LD, CVLT-II long delay; BVMT-R T1–3, average proportion accuracy on Trials 1–3; BVMT-R DR, BVMT-R delayed recall. MMQ: Multifactorial Memory Questionnaire; ^a^Sample size (*n* = 21, *n* = 29); ^b^Sample size (*n* = 20, *n* = 30); ^c^Sample size (*n* = 17, *n* = 23); ^d^Sample size (*n* = 17, *n* = 21). *p < 0.05; **p < 0.01.

**Table 4:** (A) Intercorrelations for Rank, MoCA, CVLT-II Long Delay Free Recall Proportion Accuracy (CVLT LD), and Digits Backward at baseline.**(B)** Hierarchical regression analysis summary for age, years of education (YOE), MoCA, CVLT-II Long Delay Free Recall (CVLT LD Free), Digits Forward,and Digits Backward at baseline predicting rank.

**(A)**
	**Rank**	**Age**	**YOE**	**MoCA**	**Digit Span Forward**	**Digit Span Backward**	**CVLT-II LD free**
Rank	1.00	0.311*	-0.201	-0.472**	-0.049	-0.367*	-0.398*
Age		1.00	0.251	-0.155	-0.066	-0.281	-0.247
YOE			1.00	0.266	0.189	0.038	0.240
MoCA				1.00	0.263	0.227	0.336*
Digit Span Forward					1.00	0.435**	0.067
Digit Span Backward						1.00	-0.074
CVLT-II LD							1.00

*p < 0.05; **p < 0.01.

**(B)**

**Model**	**Variable**		**B**		**SEB**	**Beta**	***p*-value**

1	Age		0.612		0.286	0.386	0.041
	YOE		-0.847		0.513	-0.297	0.110
2	Age		0.211		0.288	0.133	0.471
	YOE		-0.336		0.503	-0.118	0.511
	MOCA		-1.019		0.591	-0.303	0.098
	Digit Span Forward		1.271		0.909	0.248	0.176
	Digit Span Backward		-1.455		0.702	-0.385	0.049
	CVLT LD Free		-11.775		7.549	-0.280	0.132

*Model 1 *R*^*2*^ = 0.18; *R* Adj = 0.12 (*N* = 29, *p* = 0.069).*

*Model 2 *R*^*2*^ = 0.45; *R* Adj = 0.31 (*N* = 29, *p* = 0.022).*

In digits backward, there was a significant time effect *F*(1,48) = 9.71, *p* = 0.003, η^2^ = 0.17, but no group effect or interaction (*p*s ≥ 0.31), suggesting that both groups improved to a similar degree. (**Table 3**).

#### Transfer effects

A series of repeated measures 2 (Group) × 2 (Time) ANOVAs were run for each of the non-trained measures. Scores are shown in **Table 3**.

***California Verbal Learning Test-Second Edition.*** To reduce the chance of a Type I error, the following subscores were selected for analysis: (1) five trials of List A immediate free recall; (2) Short Delay Free Recall (3) Long Delay Free Recall (both free recall tasks were chosen over cued to avoid any influence of familiarity on recall), and (4) total across list intrusions on Long Delay Free Recall. Recognition scores were at ceiling for many people and were therefore not used in this analysis. No significant main effects or interactions were observed *p*s ≥ 0.06. (**Table 3**). Please note that one person from the recollection group was excluded from this analysis, because they had previously done one of the versions of the CVLT.

***Brief Visuospatial Memory Test-Revised.*** No time, group or interaction effects were observed (*p*s > 0.46) in the performance of participants on Trial 1–3 or on delayed recall (**Table 3**).

***Digit Span Forward and Backward.*** A significant time by group interaction on digits forward *F*(1,48) = 4.49, *p* = 0.039, η^2^ = 0.09 indicated that recollection group participants improved after training whereas recognition group participants did worse than before. There were no group or time effects (**Table 3**; *p*s ≥ 0.87).

***Source memory task.*** The only significant result among the four source memory measures was a significant group by time interaction on the spatial source memory task *F*(1,38) = 4.21, *p* = 0.047, η^2^ = 0.10. This interaction effect stemmed from the recognition group’s accuracy slightly decreasing from pre- to post-training assessment (3%), in the face of the recollection training group improving by almost 10% (**Table 3**).

***Multifactorial Memory Questionnaire.*** No main effects or interactions were observed (*p*s ≥ 0.09) in any of the three measures of the MMQ: Contentment, Ability or Strategy (**Table 3**; higher scores indicate higher levels of each measure).

#### Do those participants who show the greatest gains in training also show the greatest improvements on the transfer tasks?

We ran partial correlations to examine the relationship between rank and the change scores (between baseline and follow-up) on a selected set of transfer measures. We chose only one subscore from the CVLT-II (most subscores are highly intercorrelated)—the Accuracy Score of the Long Delay Free Recall as a classical measure of long-term memory, Digit Span Forward and Backward as measures of working memory, and the Spatial Source Memory Score (given it was the only significant source memory test). Age, and baseline scores of Digit Span Backward were controlled for in the analysis, given these variables were the only ones that were significant predictors for rank. No correlations were seen between rank and the change scores on CVLT Long Delay Free Recall, or Digit Span Forward or Backward. There was, however, a significant correlation between the change in Spatial Source Memory and rank, *r* = 0.37, *p* (one-tailed) = 0.048, *n* = 19.

## DISCUSSION

Our goal was to examine the efficacy of a “recollection training” paradigm (Jennings and Jacoby, 2003) in older adults, including possible transfer to non-trained measures of long-term and working memory. The recollection training group developed a greatly enhanced ability to reject the repeated foils in the training task, and both groups improved in recognition accuracy (d′) on their respective training tasks. Despite this, performance on near-, intermediate- and far- transfer tests was affected little by the recollection training. Individual differences in cognitive ability appeared to play a role in the training: Those participants who achieved the longest lags on the recollection training were younger and had better working memory. While not significant, better global cognitive function (as represented by MoCA scores) also seemed to be a good predictor.

### CLEAR IMPROVEMENTS ON THE TRAINING TASK

Initially, the older adults in the recollection training group made repetition errors in the training paradigm even when only a couple of items intervened between the first and second presentations of a foil. By the end of training, however, on average the group had reached a lag of 28 intervening items. These gains are commensurate with previous reports using this paradigm (Jennings and Jacoby, 2003; Jennings et al., 2005; Bailey et al., 2011). The recollection training group improved not only in the repetition error rate but also in the overall discrimination index (d′). Yet discrimination *also* improved in the recognition control group after training, which stands in contrast to previous reports (Jennings et al., 2005; Boller et al., 2012). Our control condition was more challenging than that used by Jennings et al. (2005), but yet similar to the one used by Boller et al. (2012) who also used a verbal recognition task with lists of equal length in both training and control conditions. Similar degree of improvement across treatment groups in the training task may be due merely to task practice effects (Verhaeghen et al., 1992; Engle et al., 1999; Owen et al., 2010).

### PREDICTORS OF TRAINING GAINS

Similar to previous studies (Jennings and Jacoby, 2003; Boller et al., 2012), we found considerable variability among participants in improvements on the recollection training paradigm (see **Figure 3**). Future work on this paradigm will benefit from our knowing which factors influence individuals’ training gains. When we rank ordered all the recollection training participants by their progress through the lags, we found that those who were younger progressed further through the training. Bissig and Lustig (2007) reported that age was negatively associated with training benefits in the same training procedure, whereas verbal intelligence was positively associated with it. However, we did not find a relationship between years of education (i.e., a reasonable proxy for intelligence), and rank. Past meta-analyses of memory training have produced contradictory results when it comes to the effects of age on training gains, with some reporting a significant relationship (Yesavage et al., 1990) and others not (Gross et al., 2012). A separate model including several cognitive baseline measures (MoCA, Digit Span Forward and Backward, and CVLT-II Long Delay Free Recall) improved significantly the interpretation of rank scores over and above the influence of age and years of education. Among those cognitive measures, Digit Span Backward and MoCA were the best predictors. Although one would hope to see lower functioning people (who arguably need help the most) show greater benefits of recollection training, it appears that older adults with better cognitive status and working memory might be the ones who benefit more. This so-called “Matthew effect” (in which the cognitively rich get richer following training, and the cognitively poor do not benefit as much) is evident in other cognitive training studies in older adults (Yesavage et al., 1990; Verhaeghen et al., 1992) although it is far from universal (e.g., Belleville et al., 2006).

### LIMITED EVIDENCE OF TRANSFER

The more important question regarding the potential effectiveness of the training is whether we found transfer to non-trained tasks and materials. We used the recollection training paradigm in the present study because of the previous reports of transfer in older adults (Jennings and Jacoby, 2003; Jennings et al., 2005; Bailey et al., 2011). Yet, in the present study, although both groups improved significantly over time on their respective training tasks, we observed few convincing transfer effects. The only two cases in which the recollection training group improved to a greater degree than the recognition control group were in forward digit span and the spatial subtest of the source memory paradigm. In both cases, the recollection training group’s scores increased slightly in the face of the recognition group’s scores *decreasing* after training. We would be more confident in these effects if we had additionally found that those participants who improved to a greater degree on the recollection training task also improved to a greater degree on these two transfer tests, but this was not the case. In addition, it is puzzling why we might have found an effect of recollection training on source memory for spatial location, but not on the source memory subtests for voice or temporal context, especially given that the recollection training required participants to remember *when* they saw a word and the task did not have a voice or spatial component. There is some evidence that temporal context memory may be more affected than spatial context memory by aging, which could cause them to be differentially affected by training (Parkin et al., 1995). Note also that *both* training groups improved significantly on Backward Digit Span. Whether this is a genuine training effect or merely a product of non-specific factors such as improvement in mood or comfort/reduction in stress, benefits of social/intellectual stimulation, and/or participants’ expectations of improvement, cannot yet be determined. Digit span was considered a far-transfer measure and as such least likely to show benefits. At the same time, previous recollection training studies have shown transfer to working memory measures (Jennings et al., 2005; Bailey et al., 2011; Boller et al., 2012). This suggests that this training paradigm may tap more heavily into working memory (as opposed to episodic memory) than was originally intended. This is further supported by the fact that despite the larger sample size, we still failed to see transfer to free recall in the CVLT. In addition, Digit Span Backward was the only task that significantly predicted rank in the training group. These results, along with the time by group interaction found with the Digit Span Forward task, may speak to the potential overlap between working memory and episodic memory processes, but at present it is too soon to tell. Further examination with experimental measures of recollection and working memory as transfer measures may be warranted to better delineate the two.

Why did we find only limited evidence of transfer in this study? Three possible explanations come to mind. First, any time one fails to find a predicted effect, the question of statistical power can be raised. Yet, we had an adequate sample size (*n* = 30) to detect large within-group recollection training effects, and large effects were reported by Jennings et al. (2005) and Bailey et al. (2011). Note also that in the current study we are not merely reporting null effects across the board; rather, we are reporting a clear dissociation between the recollection group’s improved ability to reject repeated foils after training and a relative lack of change on the non-trained tests. In an attempt to balance the risks of Type I versus Type II errors in our statistical analyses of the transfer test scores, we adopted the strategy of running as few repeated-measures ANOVAs as possible (to reduce the likelihood of making a Type I error), and at the same time keeping our alpha at 0.05 (to reduce the likelihood of making a Type II error). Yet, even if we were to use a much more liberal statistical threshold (e.g., *p* = 0.10), none of the other Group × Time interactions in **Table 3** would become significant.

Second, although we used a training schedule that was similar or identical to previous reports (Jennings and Jacoby, 2003; Jennings et al., 2005; Bailey et al., 2011; Boller et al., 2012), the participants might have shown more robust transfer effects if we had increased the intensity (i.e., the “dose”) or the duration of training. Note, however, that a relative advantage of the current study was that all of our participants completed the training. Increasing the intensity, frequency, or length of training would increase the danger of a selective sample being recruited, and of at least some participants dropping out during training, which would complicate the interpretation of the data.

Third, the effects of our particular recollection training method might be restricted to specific cognitive processes. The repetition lag paradigm that we used for recollection training involved monitoring visually presented words during a yes-no recognition memory test to make a decision regarding the list membership (study versus test list) of each item. Although we employed two “near” transfer tests that we thought were very much akin to the repetition lag training task, these might not have been similar enough. It might be that only very precise test format-, modality-, and/or stimulus-specific gains should be particularly evident after training with the current protocol. For example, using this repetition lag protocol, Jennings et al. (2005) and Boller et al. (2012) *did* find a benefit of recollection training on source memory. In our study, we only found a significant transfer effect to Spatial Source Memory and these scores also served as good predictors of the gains made in the training as measured by rank. One key difference between studies may be that whereas we presented our source memory materials auditorily, the previous two studies presented theirs visually. We also used full sentences, while previous studies used single words/pictures. Similarly, in a very recent study, McDaniel et al. (2014) used Jennings and Jacoby’s (2003) recollection training procedure as one of three components of a cognitive training program Older adults were trained with words as stimuli, but to examine transfer they completed a very similar task that incorporated repeated lures using sentences. Further, each sentence was presented in a specific context, which should have increased the contextual detail available for recollection of the stimulus. However, the authors failed to observe any transfer to this task despite its similarity to the actual training procedure. This further supports the notion that the benefits of the recollection-training procedure may have a limited ability to transfer to stimuli outside of those that were trained. If the benefits of recollection training do turn out to be relatively idiosyncratic, as found here, then the impact of this recollection training method on memory in everyday life will be limited.

Finally, on a positive note, we observed that after training, our participants did *not* alter their subjective ratings of memory Ability or Contentment on the MMQ (Troyer and Rich, 2002), which is in keeping with their lack of improvement on the objective tests they were given. The lack of change in subjective measures of memory is beneficial, since we do not want participants to *think* they have improved substantially when in fact they have not. Some cognitive training methods in the past have reported improvements in subjective memory ratings in both control and treatment groups (Berg et al., 1991). A necessity for any potential cognitive training method though is that it does not overinflate confidence.

## CONCLUSION AND FUTURE DIRECTIONS

In summary, we found relatively weak transfer effects of recollection training, despite quite significant gains in performance on the training task itself. Two sets of questions must be addressed to allow progress with this (and other) cognitive training methods.

The first set of questions concerns the training itself. As outlined above, a more nuanced assessment of the processes affected by recollection training might yield clearer transfer effects. In addition, notwithstanding the problems inherent to increasing the dose or the duration of training, if one were able to use this training method over the long term, a different picture might emerge: That is, whereas the present study focused on potential *improvements* in memory performance over the relatively short term, if such training could be implemented over the long term it might help to stave off memory *decline* over time. For example, a more intensive training program with booster sessions, the ACTIVE study, has shown effects on reasoning and speed of processing that have persisted over a decade, and are associated with fewer self-reported difficulties in Instrumental Activities of Daily Living (Rebok et al., 2014), which are important for maintaining functional independence with age. If one wanted to address this question with the current training approach, drop-out could probably be attenuated by altering the paradigm (for example, by performing the training at home, rather than requiring frequent visits to the lab). Finally, combining recollection training with neurophysiological, pharmacological, or other behavioral therapies might yield clearer effects on everyday memory activities (Ranganath et al., 2011).

The second set of questions concerns who should receive (and who might benefit from) such training. In the present study, higher-functioning older adults improved to a greater degree than did lower-functioning ones on the recollection training task itself (although this had little bearing on the transfer measure scores). Might there be a way to modify the paradigm so that it aids low-functioning older adults to a greater extent? For example, Boller et al. (2012) modified the recollection-training task to make it more suitable for Alzheimer’s patients. They shortened the length of the word lists, increased the length of presentation of the words and lowered the maximum lag levels (albeit no patients reached the maximum lag level). Other modifications could include lengthening the encoding interval in a similar fashion as that done by Lustig and Flegal (2008). Modifications like these could prove to be more effective for lower functioning older adults, because they might allow the task to be more manageable and yet still challenging.

## Conflict of Interest Statement

The authors declare that the research was conducted in the absence of any commercial or financial relationships that could be construed as a potential conflict of interest.
