# Corrigendum: Training Recollection in Healthy Older Adults: Clear Improvements on the Training Task, but Little Evidence of Transfer

**DOI:** 10.3389/fnhum.2015.00658

**Published:** 2015-12-01

**Authors:** Vessela Stamenova, Janine M. Jennings, Shaun P. Cook, Lisa A. S. Walker, Andra M. Smith, Patrick S. R. Davidson

**Affiliations:** ^1^Rotman Research Institute, Baycrest – University of TorontoToronto, ON, Canada; ^2^Department of Psychology, Wake Forest UniversityWinston-Salem, NC, USA; ^3^Department of Psychology, Millersville UniversityMillersville, PA, USA; ^4^School of Psychology, Faculty of Social Sciences, Ottawa Hospital Research Institute, University of OttawaOttawa, ON, Canada

**Keywords:** aging, memory, rehabilitation, recollection, familiarity

Due to errors that were noticed recently in the California Verbal Learning Test-II (CVLT-II) scoring software version 1.0.0 with the software occasionally mis-counting items, we have recalculated all CVLT scores and re-run all relevant analyses. This update was completed with a software update (to version 1.0.2) (see http://pearsonassessmentsupport.com/support/index.php?View=entry&EntryID=741).

The majority of the statistical effects remain the same as in the original publication, except for two.

These are:

Section: ***Predictors of training gains***

1. For the regression, the initial analysis showed that adding the additional variables led to a significant increase in prediction. Now this result is ***marginally*** significant, at *p* = 0.056. The initial increase was by 45% and now it is by 44%.
2. In the same regression, Model 2 Digit Span Backward was initially statistically significant at *p* = 0.049, but after corrections now *p* = 0.058. We state that this effect is ***marginally*** significant. Because of this initially significant effect, in a partial correlation conducted later on section entitled: ***Do those participants who show the greatest gains in training also show the greatest improvements on the transfer tasks?***, we controlled for Digit Span backwards scores. We chose to still control for these scores, given that the significant test is so close to the cut-off.

No other effect changes were observed in any of our analyses involving the CVLT. The values, however, have changed slightly and we have updated those values in Tables 3, 4 of the manuscript.

**Table 3:** **Pre- and post-training scores on transfer measures**.

	**Recognition control**		**Recollection training**		***p*****-value**		
	**Pre**	**Post**	**Pre**	**Post**			
	***M (SD)***	***M (SD)***	***M (SD)***	***M (SD)***	**Group**	**Time**	**Group × Time**
CVLT-II List 1-5 proportion accuracy^a^	0.77 (0.10)	0.76 (0.13)	0.70 (0.15)	**0.72 (0.12)**	**0.11**	**0.86**	**0.49**
CVLT-II SD free proportion accuracy^a^	**0.80 (0.16)**	**0.81 (0.18)**	0.71 (0.22)	**0.75 (0.21)**	**0.15**	**0.23**	**0.43**
CVLT-II LD free proportion accuracy^a^	**0.76 (0.24)**	**0.81 (0.26)**	**0.76 (0.21)**	**0.78 (0.23)**	**0.86**	**0.50**	**0.50**
CVLT-II total across list intrusions	**0.01 (0.02)**	**0.05 (0.09)**	**0.05 (0.12)**	**0.06 (0.15)**	**0.24**	**0.15**	**0.38**
BVMT-R T1-3 proportion accuracy	0.69 (0.21)	0.71 (0.21)	0.71 (0.11)	0.72 (0.13)	0.70	0.46	0.97
BVMT-R DR proportion accuracy	0.83 (0.15)	0.84 (0.14)	0.83 (0.13)	0.83 (0.13)	0.94	0.73	0.95
Digit Span Forward^b^	10.60 (2.01)	10.20 (1.40)	10.23 (1.72)	10.70 (1.74)	0.89	0.87	0.04^*^
Digit Span Backward^b^	7.45 (2.42)	8.90 (2.36)	7.23 (2.33)	7.97 (2.63)	0.35	0.003^**^	0.31
Source memory spatial %^c^	62.50 (15.31)	59.19 (14.20)	58.15 (13.25)	67.39 (12.10)	0.54	0.34	0.047^*^
Source memory temporal %^d^	66.91 (15.11)	64.33 (11.43)	63.10 (13.67)	65.77 (17.52)	0.09	0.99	0.36
Source memory voice %^d^	72.79 (16.82)	77.57 (17.12)	75.60 (13.82)	73.51 (17.66)	0.89	0.61	0.20
Source memory item %^d^	94.26 (4.40)	93.24 (7.22)	92.07 (9.22)	90.87 (10.99)	0.37	0.35	0.94
MMQ contentment (/72)	39.33 (12.08)	42.38 (10.77)	40.73 (12.00)	41.73 (9.87)	0.90	0.10	0.40
MMQ ability (/80)	46.1 (9.20)	47.60 (9.28)	48.00 (10.78)	49.41 (10.72)	0.49	0.24	0.97
MMQ strategy (/76)	41.10 (8.74)	38.0 (7.21)	35.93 (10.92)	35.20 (8.31)	0.09	0.14	0.36

*CVLT-II List 1-5, Average proportion accuracy on Trials 1-5; CVLT-II SD, CVLT-II Short Delay; CVLT-II LD, CVLT-II Long Delay; BVMT-R T1-3, Average proportion accuracy on Trials 1-3; BVMT-R DR, BVMT-R Delayed Recall. MMQ, Multifactorial Memory Questionnaire*.

a *Sample size (n = 21, n = 29)*.

b *Sample size (n = 20, n = 30)*.

c *Sample size (n = 17, n = 23)*.

d *Sample size (n = 17, n = 21)*.

* p < 0.05;

** *p < 0.01*.

Table 4**Intercorrelations for Rank, MoCA, CVLT-II Long Delay Free Recall Proportion Accuracy (CVLT LD), and Digits Backward at baseline**.

**Table:** 

	**Rank**	**Age**	**YOE**	**MoCA**	**Digit Span Forward**	**Digit Span Backward**	**CVLT-II LD Free**
Rank	1.00	0.311^*^	–0.201	–0.472^**^	–0.049	–0.367^*^	–0.392^*^
Age		1.00	0.251	–0.155	–0.066	–0.281	**–0.225**
YOE			1.00	0.266	0.189	0.038	**0.259**
MoCA				1.00	0.263	0.227	0.353^*^
Digit Span Forward					1.00	0.435^**^	**0.058**
Digit Span Backward						1.00	**–0.059**
CVLT-II LD							1.00

*Hierarchical Regression Analysis summary for Age, Years of Education (YOE), MoCA, CVLT-II Long Delay Free Recall (CVLT LD Free), Digits Forward, and Digits Backward at baseline predicting rank*.

* p < 0.05

** *p < 0.01*.

**Table:** 

**Model**	**Variable**	**B**	**SEB**	**Beta**	***p*****-value**
1	Age	0.612	0.286	0.386	0.041
	YOE	–0.847	0.513	–0.297	0.110
2	Age	**0.236**	**0.287**	**0.149**	**0.421**
	YOE	**–0.346**	**0.509**	**–0.122**	**0.503**
	MOCA	**–1.024**	**0.600**	**–0.305**	**0.101**
	Digit span forward	**1.231**	**0.917**	**0.240**	**0.192**
	Digit span backward	**–1.403**	**0.703**	**–0.371**	**0.058**
	CVLT LD Free	**–10.800**	**7.627**	**–0.256**	**0.170**

*Model 1. R^2^ = 0.18, R Adj = 0.12 (N = 29, p = 0.069)*.

***Model 2. R^2^ = 0.44, R Adj = 0.29 (N = 29, p = 0.026)***.

Overall, given the changes affect only one of our outcome measures and the fact that the results with this outcome measure have changed only for two statistical tests, we believe that the correction does not affect the scientific validity of the results.

Please see below the relevant changes made, with bold font for the edited or inserted text.

***In section “Predictors of Training Gains,”***

The regression results are summarized in Table 4; Multiple R for the first block of regressors (age and YOE) was close to statistical significance, *F*_(2, 29)_ = 2.96, *p* = 0.069; multiple R for the next block of regressors was significant *F*_(6, 29)_ = **3.00**, ***p* = 0.026**. The demographic variables (Age and YOE) explained 18% of the variance, while adding the cognitive status scores in block 2 of the analysis increased the amount of variability explained to **44**%. This increase is **marginally** significant by the *F* change test ***F***_**(4, 23)**_ **= 2.66**, ***p*** **= 0.056**. Among the demographic variables, only age was significant, while among the cognitive status, Digit Span Backwards (***p*** **= 0.058**) was **marginally** statistically significant, followed by the MoCA (*p* = **0.101**), which was marginally significant.

***In section “Do those participants who show the greatest gains in training also show the greatest improvements on the transfer tasks?,” last sentence:***

There was, however, a significant correlation between the change in Spatial Source Memory and rank, ***r*** = **–0.37, *p* (one-tailed)** = **0.048, *df*** = **19**.

Please see below relevant changes made to Tables 3, 4.

## Conflict of interest statement

The authors declare that the research was conducted in the absence of any commercial or financial relationships that could be construed as a potential conflict of interest.

